# Complete hemispheric exposure vs. superior sagittal sinus sparing craniectomy: incidence of shear-bleeding and shunt-dependency

**DOI:** 10.1007/s00068-021-01789-8

**Published:** 2021-10-04

**Authors:** Martin Vychopen, Matthias Schneider, Valeri Borger, Patrick Schuss, Charlotte Behning, Hartmut Vatter, Erdem Güresir

**Affiliations:** 1 Department of Neurosurgery, University Hospital Bonn, Venusberg Campus 1, 53127 Bonn, Germany; 2 Department of Medical Biometry, Informatics and Epidemiology, Universität Bonn, Institut für Medizinische Biometrie, Informatik und Epidemiologie (IMBIE), Bonn, Germany

**Keywords:** Decompressive hemicraniectomy, Size, Shear-bleeding

## Abstract

**Purpose:**

Decompressive hemicraniectomy (DC) has been established as a standard therapeutical procedure for raised intracranial pressure. However, the size of the DC remains unspecified. The aim of this study was to analyze size related complications following DC.

**Methods:**

Between 2013 and 2019, 306 patients underwent DC for elevated intracranial pressure at author´s institution. Anteroposterior and craniocaudal DC size was measured according to the postoperative CT scans. Patients were divided into two groups with (1) exposed superior sagittal sinus (SE) and (2) without superior sagittal sinus exposure (SC). DC related complications e.g. shear-bleeding at the margins of craniectomy and secondary hydrocephalus were evaluated and compared.

**Results:**

Craniectomy size according to anteroposterior diameter and surface was larger in the SE group; 14.1 ± 1 cm vs. 13.7 ± 1.2 cm, *p* = 0.003, resp. 222.5 ± 40 cm^2^ vs. 182.7 ± 36.9 cm^2^, *p* < 0.0001. The SE group had significantly lower rates of shear-bleeding: 20/176 patients; (11%), compared to patients of the SC group; 36/130 patients (27%), *p* = 0.0003, OR 2.9, 95% CI 1.6–5.5.

There was no significant difference in the incidence of shunt-dependent hydrocephalus; 19/130 patients (14.6%) vs. 24/176 patients (13.6%), *p* = 0.9.

**Conclusions:**

Complete hemispheric exposure in terms of DC with SE was associated with significantly lower levels of iatrogenic shear-bleedings compared to a SC-surgical regime. Although we did not find significant outcome difference, our findings suggest aggressive craniectomy regimes including SE to constitute the surgical treatment strategy of choice for malignant intracranial pressure.

## Backround

Decompressive hemicraniectomy (DC) is an established surgical method for treatment of raised intracranial pressure caused by cerebral infarction (CI) [1], traumatic brain injury (TBI) [2], subarachnoid hemorrhage (SAH) [3] and intracerebral hemorrhage (ICH) [4]. An adequate anteroposterior diameter of DC has already been described to be *at least 12 cm,* accompanied with adequate temporobasal decompression [5, 6]. Contrary to AP diameter [7]; the optimal craniocaudal diameter remains unspecified. An agressive craniectomy, including maximal decompression with exposure of superior sagittal sinus, might be a risk factor for shunt-dependency. This study aims to analyze the size related complications of two different surgical techniques of DC according to anatomical landmarks: patients undergoing complete hemispheric exposure vs. those without exposure of the superior sagittal sinus (SSS). We mainly focused on the incidence of shunt-dependent hydrocephalus and the incidence of shear-bleeding at the edge of DC.

## Methods

**Fig. 1:**
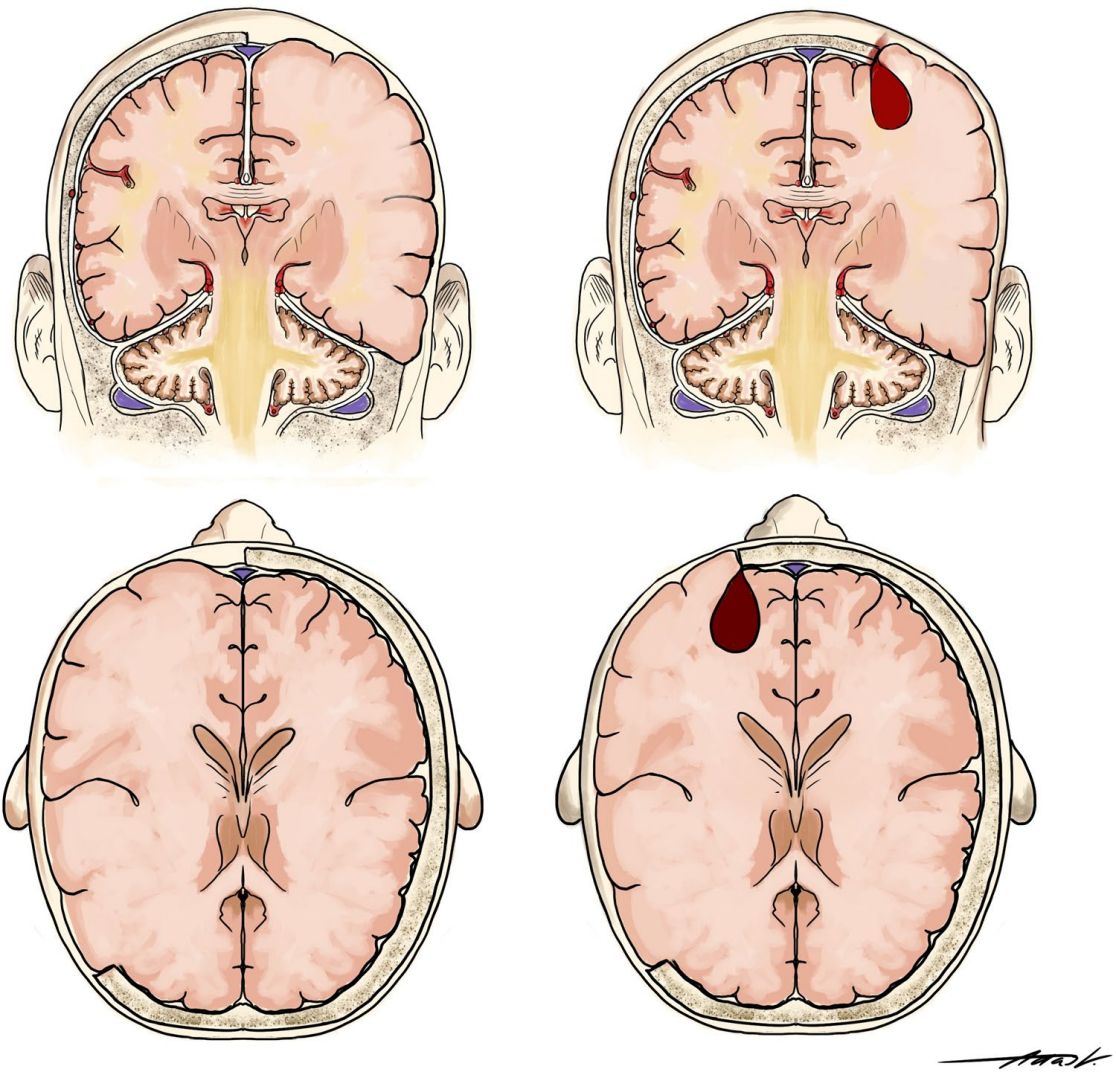
Left—coronar and axial view of the SE group. Right—coronar and axial view of the SC group

**Fig. 2:**
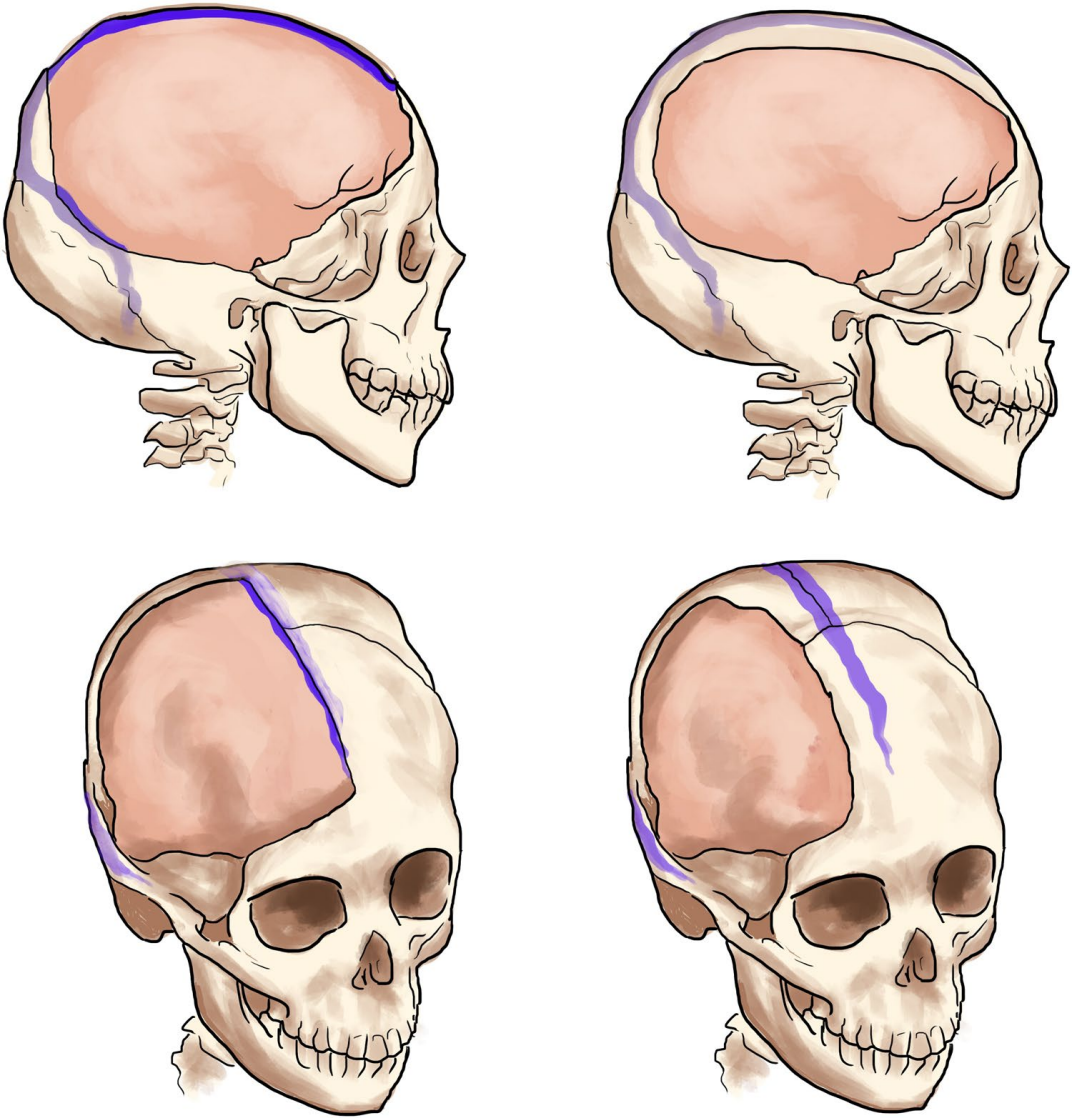
Left—3D view of SE group. Right—3d view of SC group

A retrospective single center study of patients undergoing DC from 02/2013 to 10/2019 was performed. The extent of the craniectomy was analyzed using early postoperative CT scans with DICOM Viewer software. We measured the anteroposterior diameter as proposed by Flint et al. [8]. The surface of the craniectomy was calculated using the De Bonis equation [9]. We divided the patients in two groups based on anatomical landmarks: (1) patients with complete exposure of superior sagittal sinus (SE) and (2) patients without exposure of superior sagittal sinus (SC) (Figs. 1 and  2).

### Surgical techniques

The head of the patient is positioned with his head rotated parallel to the floor, the side of the craniectomy pointing upwards. Mayfield skull clamp is used to fix the head. By unilateral decompressive hemicraniectomy, the half of the head is shaved. The incision in the shape of a reverse question mark is starting at the tragus and continuing slightly across the midline. The trauma flap is created [10].

*Group 1 (SE)*—after exposing the skull, sagittal suture is identified and two burrholes are placed on the sagittal suture, where SSS is expected, determining the medial craniectomy edge. The sagittal sinus was exposed by craniectomy on sagittal suture. Usually, full exposure of the hemisphere including the exposure of the SSS is reached. Duraplasty is not performed.

*Group 2 (SC)*—after identifying the sagittal suture, the burrholes are placed ipsilateral on the site of the craniectomy, leaving 1–2.5 cm distance between the edge of the craniectomy and sagittal suture. Both sinus and bridging veins are covered with bone. Duraplasty is also not performed.

The sinus exposure was performed according to attending neurosurgeon.

### Peri/postoperative complications

#### Shear-bleeding

**Fig. 3:**
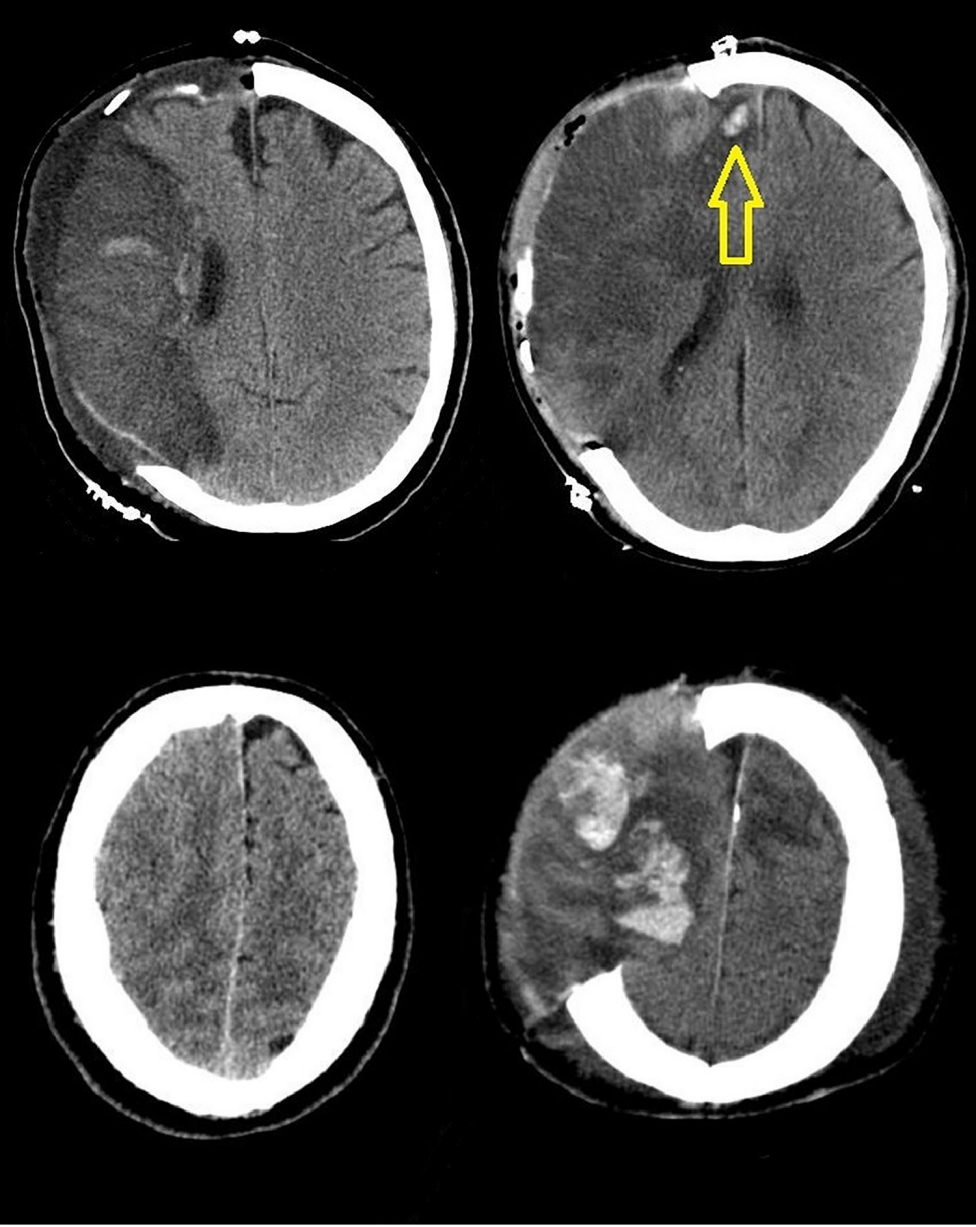
Above left—axial CT scan of patient from SE group. Above right—axial CT scan of patient from SC group, the arrow is pointing on shear-bleeding. Below left—preoperative CT scan of patient from SC group. Below right—postoperative CT scan with newly developed shear-bleeding

All available postoperative CT scans were analyzed for the incidence of newly developed intraparenchymal hemorrhage at the edge of the craniectomy (Fig. 3).

#### Shunt-dependent hydrocephalus

The focus was given on patients who developed shunt-dependent hydrocephalus after DC and underwent secondary shunt implantation.

#### Further postoperative complications

We retrospectively analyzed and compared perioperative blood-loss, the need of red blood cell transfusions, wound healing disturbances, operating time, air embolism, sinus thrombosis and intraoperative sinus injury. We divided the outcome of the patients as favorable (mRS ≤ 3) and unfavorable (mRS > 3).

### Statistics

A Fisher’s exact test was used to demonstrate the association between the sinus exposure and development of shear-bleeding and shunt-dependent hydrocephalus. Values with *p* < 0.05 were considered to be significant. Finally, propensity score adjustment was performed to analyze the risk-bleeding risk factors between the groups.

## Results

### Patient characteristics

381 patients underwent DC at our institution between 02/2013 and 10/2019. 75 patients were excluded because of: insufficient postoperative imaging (*n* = 43), bifrontal craniectomy (*n* = 17) and age < 18 years (*n* = 15). 306 patients were included in the analysis, 176 in SE group and 130 in SC group (see Table 1). The underlying diagnosis was TBI (*n* = 85), ICH (*n* = 68), CI (*n* = 81), SAH (*n* = 47) and miscellaneous pathologies including meningitis (*n* = 4), postoperative edema of unclear origin (*n* = 13), sinus thrombosis (*n* = 4), spontaneous subdural hematoma (*n* = 1), bleeding after electrode implantation (*n* = 1), cerebral edema after callosotomy (*n* = 1) and primary cerebral edema of unclear origin (*n* = 1). For details, see Table 1*.*

**Table 1:** Patient characteristics

Sex	
Male	171
Female	135
Underlying pathology	
TBI	85 (27.7%)
ICH	68 (22.2%)
CI	81 (26.5%)
SAH	47 (15.4%)
Miscellaneous pathologies	25 (8.1%)
Mean age (± SD) in years	57.26 ± 15.54
Number of patients	306
SE group	176 (57.5%)
SC group	130 (42.5%)

*TBI* traumatic brain injury, *ICH* intracerebral hemorrhage, *CI* cerebral infarction, *SAH* subarachnoid hemorrhage, *SD* standard deviation, *SE group* sinus exposed group, *SC group* sinus not exposed group

### Anteroposterior diameter and surface

Anteroposterior diameter of the DC was overall of 13.7 cm ± 1.2 cm. The AP Diameter was significantly larger in SE Group compared to SC Group; 14.1 ± 1.1 cm vs. 13.7 ± 1.2 cm, *p* = 0.003.

There was a significant difference in surface of the resected bone between both groups; SE Group 222.5 ± 40.0 cm^2^ vs. SC Group 182.7 ± 36.9 cm^2^, *p* < 0.0001. For detailed information, see Table 2*.*

**Table 2:** Sinus exposed vs. sinus covered - group analysis

	SE group (*n* = 176)	SC group (*n* = 130)	
*Men:women*	*95 (53.9%):81 (46.1%)*	*80 (61.5%):50 (38.5%)*	*p* = 0.18
*INR*	*1.2* ± *0.4*	*1.1* ± *0.4*	*p* = 1.0
*Platelets (G/l)*	*228* ± *98.5*	*238* ± *102.79*	*p* = *0.4*
*aPTT (s)*	*26* ± *8.5*	*25* ± *4.9*	*p* = *0.2*
*Hemoglobin (g/dl)*	*12.4* ± *2.2*	*12.6* ± *2.09*	*p* = *0.4*
*History of anticoagulants*	*43/176 (24.4%)*	*32/130 (24.6%)*	
*GCS at admission*	*8* ± *4.42*	*8.5* ± *4.29*	*p* = *0.3*
*Operative time (min)*	*86.3* ± *26.6*	*99.3* ± *31.8*	*p* = *0.0001*
AP-diameter	14.1 ± 1.1 cm	13.19 ± 1.1 cm	*p* = 0.0027
Surface of DC	222.50 ± 40.0 cm^2^	182.7 ± 36.9 cm^2^	*p* < 0.0001
Shear-bleeding	20 (11%)	36 (27%)	*p* = 0.0003
Shunt-dependency	24 (13.6%)	19 (14.6%)	*p* = 0.9
Wound-healing disturbances	12	7	*p* = 0.607
Blood loss ≤ 500 ml	47 (26.7%)	26 (26.7%)	
Red blood cell transfusions	90 (51.1%)	64 (49.2%)	*p* = 0.741
Shear-bleeding localization			
Medial DC margin	11 (6.2%)	27 (20.8%)	*p* = 0.0001
Medial + lateral DC margin	3 (1.7%)	7 (5.3%)	
Lateral DC margin	6 (3.4%)	2 (4.6%)	

Italic values indicates statistically significant *p* < 0.05

*In exploratory analysis, the craniectomy type seems to be the only statistically significant factor associated with the incidence of shear-bleeding* (Table 3).

**Table 3:** Multivariate analysis of shear-bleeding risk factors

	CI 95%	*p* value
AP-diameter	0.98–1.01	0.34
Operative time	0.998–1.01	0.13
Surface	1.0–1.0	0.53
Type of craniectomy	0.22–0.85	0.01

*AP-diameter* anteroposterior diameter

### Shear-bleeding incidence and localization

20 out of 176 (11%) patients in SE group had shear-bleeding, whereas, 36 out of 130 (27%) of the patients in SC group had shear-bleeding; *p* = 0.0003, OR 2.9, 95% CI 1.6–5.5.

**Fig. 4:**
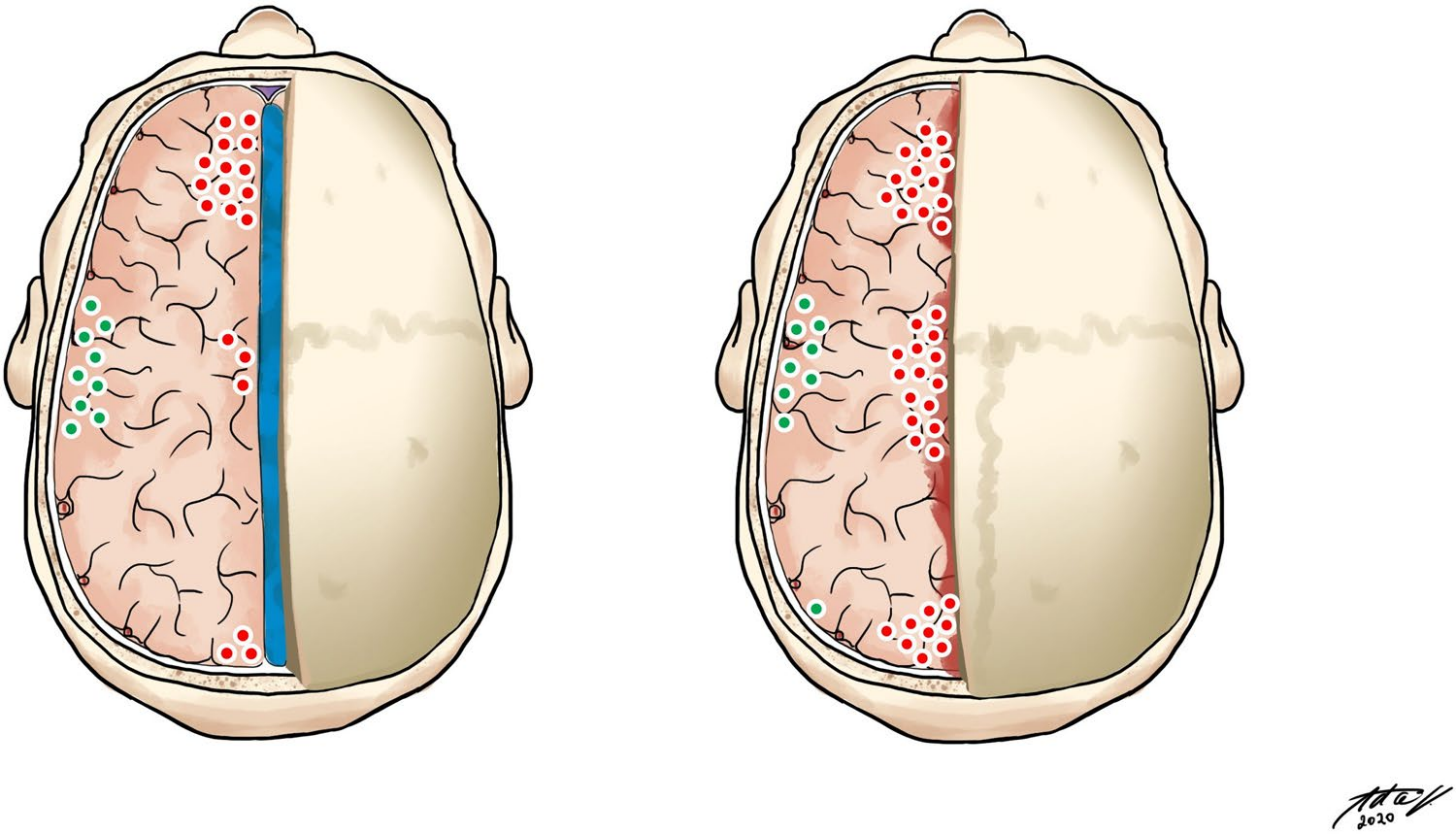
Left—shear-bleeding localization in SE group. Right—shear-bleeding localization in SC group

Most of the shear-bleeding lesions were localized near the medial boundary of the craniectomy. The incidence of these medially localized lesions was significantly lower in SE group with 6.8% vs. 20.8% in SC group; *p* = 0.0003 (Fig. 4).

#### Underlying diagnosis

**Fig. 5:**
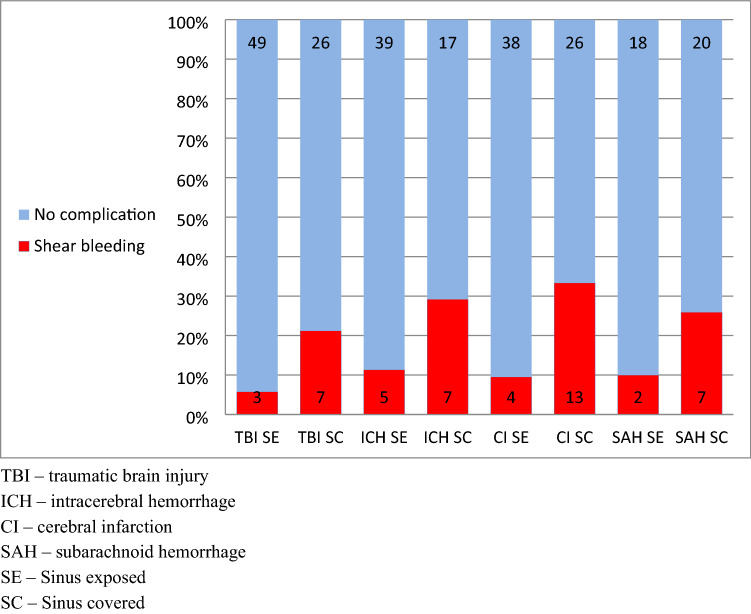
Shear bleeding according to underlying condition

SC was associated with higher incidence of shear-bleeding in all underlying conditions leading to DC. The limitation of this analysis is the low number of patients in each subgroup. See Fig. 5 and Table 4

**Table 4:** Subgroup analysis of complications

	TBI SE	TBI SC	ICH SE	ICH SC	CI SE	CI SC	SAH SE	SAH SC
No complication	**46**	**23**	**36**	**13**	**37**	**25**	**12**	**16**
Complications	**6**	**10**	**8**	**11**	**5**	**14**	**8**	**11**
Shunt	3	3	3	4	1	1	6	4
Shunt and shear-bleeding	0	1	1	1	0	2	0	2
Shear-bleeding	3	6	4	6	4	11	2	5

Bold values indicates statistically significant *p* < 0.05

*TBI* traumatic brain injury, *ICH* intracerebral hemorrhage, *CI* cerebral infarction, *SAH* subarachnoid hemorrhage, *SE* sinus exposed, *SC* sinus covered

### Shunt-dependency

43 patients developed secondary drainage-dependent hydrocephalus and underwent a shunt implantation. Shunt-dependency rates did not significantly differ between the two groups: 24 patients (13.6%) in SE group vs. 19 patients (14.6%) in SC group (*p* = 0.9).

### Intraoperative blood-loss

None of the analyzed groups presented itself with higher intraoperative bleeding volume. The blood loss was lower as 500 ml in 26.7% of cases.

The red blood cell transfusion rates did not differ between both groups; SE 90/176 (51.1%) vs. SC 64/130 (49.2%), *p* = 0.7.

### Wound-healing disturbances

Wound-healing disturbances were observed in *19 cases.* Four of them underwent the DC because of CI (2 SE vs. 2 SC), four because of SAH (2 SE vs. 2 SC) eight because of TBI (4 SE vs. 3 SC) and four because of ICH (4 SE vs. 0 SC). There was no difference between both groups noted, *p* = 0.6.

### Operative time

The operative times of the SE group were significantly shorter compared to the SC group (SE 86.3 ± 26.6 min vs. SC 99.3 ± 31.8 min, CI 95% 6.4–19.6, *p* = 0.0001). However, the mean difference was 13 min.

#### Propensity score adjustment

**Fig. 6:**
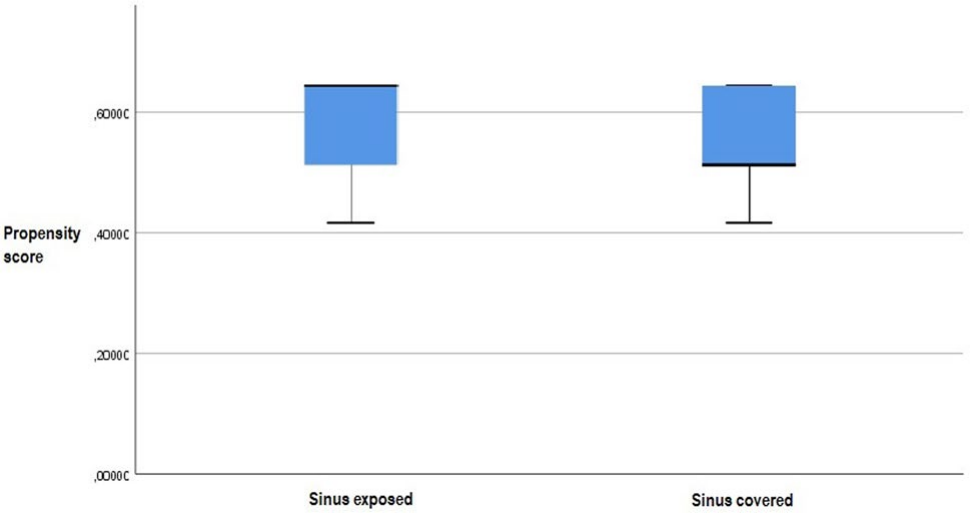
Propensity score Box-plot, sinus exposed vs. sinus covered

Propensity score analysis was performed to evaluate the shear-bleeding risk-factor. According to risk factors published by Hanko et al. [11] we included platelet count, INR, Hb, blood-thinners history and underlying pathology. *Propensity scores were 0.59* ± *0.09 in the SE Group and 0.55* ± *0.09 in the SC Group; p* = *0.003). The difference between the groups was observed in the distribution of CI (p* = *0.043, CI 0.34–0.99) and SAH (p* = *0.005, CI 0.20–0.75)* (Fig. 6).

In the logistic regression with shear-bleeding as dependent variable, the type of craniectomy as well as the propensity score as independent variables. The type of craniectomy remained significantly associated (Table 5).

**Table 5:** Propensity score vs. type of craniectomy analysis

	*p* value	CI 95%
Type of craniectomy	** < *0.0001***	***0.18–0.61***
Propensity score	***0.96***	***0.03–30.38***

Bold italic values indicate statistically significant *p* < 0.05

*CI* confidence interval

### Clinical outcome (mRS) 6 months postoperatively

Favorable outcome was observed by 37/176 (21.0%) patients in SE group vs. 30/130 in SC group (23.0%); *p* = 0.9. The analysis of the shear-bleeding subgroup showed favorable outcome by 6/20 patients in SE group (30.0%) vs. 7/36 patients (19.4%) in SC group.

## Discussion

We retrospectively analyzed patients that had undergone DC in the course of surgical treatment of pathologically raised intracranial pressure at our institution. We compared two surgical approaches of craniectomies with or without exposure of SSS. The SE group showed significantly lower incidence of shear-bleeding. No difference in shunt-dependency was noted between the groups.

### Size—anteroposterior diameter and surface of the DC

The idea of a positive correlation between size and ICP reduction was already demonstrated experimentally [12]. The size of the DC seems to play a crucial role in mortality [13] and outcome [14] by patients with TBI. The anteroposterior diameter of all DCs in our cohort exceeded the 12 cm proposed by Wagner et al. [5] as sufficient decompression. As we expected, the SE group showed significantly higher anteroposterior decompression.

Both groups overreached the DC surface size published either by De Bonis (162 cm^2^) [15] and Sturiale (168 cm^2^) [16] or by Reid (119 cm^2^) [17].

### Shear-bleeding

Our study confirmed the experimentally discussed association between the parenchyma injury around the craniectomy edges and the size of the DC [18]. We observed higher rates of shear-bleedings localized medially in the SC group. As demonstrated above, centrally localized shear-bleeding can lead to severe clinical symptomatic.

Compared to Wagner et al. [5], we did not include the patients with the increased size of ICH.

We did not classify the progression of already presented ICH as a shear-bleeding, but as “relief-effect” bleeding. “Relief-effect” bleeding is not a complication corresponding directly to the size of the DC. It is rather associated with the procedure itself as a result of sudden pressure relief during the DC, loss of tamponade effect and rapid expansion of the cerebral parenchyma [7, 19].

As expected, the SE group showed significantly lower incidence of the shear-bleeding than the SC group.

### Shunt-dependency

DC has been previously reported to be a risk factor for hydrocephalus development. The idea suggested by De Bonis et al. [15] that the incidence of shunt-dependent hydrocephalus increases if the SSS and the bridging veins are exposed has not been confirmed in our cohort. We observed the same distribution of shunt-dependency among both groups regardless of the DC size. The incidence of shunt implantation corresponds to results already reported by other authors reaching from 14.8 to 22.5% according to underlying pathology [14, 20, 21].

The proposed mechanism of hydrocephalus by DCs going near to the midline is the interference with pulsatile CSF resorption [22]^.^ DCs being “too big” are reported to interfere with the CSF flow and the occurrence of post-DC hydrocephalus signs are described in 88% of such cases [23]. In our cohort, the exposure of SSS was not accompanied with elevated level of shunt-dependency.

The discrepancies between the incidences of hydrocephalus already reported by other authors (TBI—24% [24], CI—29% [25], ICH—15–20% [26], SAB—20–35% [27]) and the incidence in our cohort may be caused by the fact that patients with radiologically presented signs of hydrocephalus who did not underwent the shunt implantation were not considered to have a shunt-dependent CSF circulation disturbance. The shunt-dependency in our cohort was; TBI—8.2%, CI—4%, ICH—8.8%, SAB—25.5%.

### Limitations

The study has several limitations. Data acquisition was retrospective, based on a single-center experience. Furthermore, non-randomized setting left the decision about the DC-size on the attending neurosurgeon. The statements of clinical outcome are limited by heterogeneous cause and seriousness of the underlying diagnosis that lead to the DC [18].

## Conclusions

Complete hemispheric exposure, and therefore larger DC size, seems to be associated with smaller likelihood of shear-bleeding, without the elevation of the incidence of procedure-related complications.

## Data Availability

Data available on request due to privacy/ethical restrictions.
